# Effects of intra-articular tranexamic acid injection with different acting times after anterior cruciate ligament reconstruction: a cohort study with historical controls

**DOI:** 10.1186/s10195-025-00826-1

**Published:** 2025-03-08

**Authors:** Kun-Han Lee, Kun-Hui Chen, Hsuan-Hsiao Ma, Tai-Jung Huang, Hsiao-Li Ma, En-Rung Chiang

**Affiliations:** 1 Department of Orthopaedics and Traumatology, Taipei Veterans General Hospital, No. 201, Sec. 2, Shih-Pai Road, Beitou District, Taipei, Taiwan 112 R.O.C; 2 School of Medicine, National Yang Ming Chiao Tung University, Taipei, Taiwan

**Keywords:** Anterior cruciate ligament reconstruction, International Knee Documentation Committee functional score, Tranexamic acid, Postoperative drainage output, Visual analog scale

## Abstract

**Background:**

Intra-articular tranexamic acid (TXA) has been proven effective in reducing postoperative bleeding in anterior cruciate ligament reconstruction (ACLR). We aimed to evaluate the effect of intra-articular injection of TXA with different acting times after an ACLR procedure.

**Patients and methods:**

Patients receiving ACLR and intra-articular injection of TXA between September 2023 and January 2024 were randomly divided into two groups, with drainage clamped for 4 h (TXA 4 h group) or 8 h (TXA 8 h group). Postoperative drainage output was the primary outcome. The secondary outcomes included the visual analog scale (VAS), grade of hemarthrosis, and International Knee Documentation Committee (IKDC) functional score. The data of another two groups of patients (TXA 2 h group and placebo group) were retrieved from a previous study as historical control groups for subsequent analysis.

**Results:**

121 patients were included. There were no significant differences in drainage output between TXA 4 h and TXA 8 h groups. On postoperative day 3, significantly decreased grades of hemarthrosis were noted in the TXA 8 h group (*P* = 0.030). There were no significant differences in the VAS at different postoperative time points or in the IKDC scores. Comparison with the placebo and TXA 2 h groups revealed significant reduction in postoperative drainage among the TXA 4 h and 8 h groups. The IKDC scores were significantly worse in the TXA 8 h group compared with the TXA 2 h (*P* < 0.001) and placebo (*P* = 0.009) groups.

**Conclusions:**

A 4 h clamping time for intra-articular TXA administration after ACLR may be considered in current practice, as it effectively reduces drainage and pain without negatively impacting functional outcomes.

**Level of evidence:**

Level III, cohort study.

## Introduction

Tranexamic acid (TXA) is a synthetic analog of the amino acid lysine, functioning as an antifibrinolytic agent. It competitively inhibits the lysine-binding sites in plasmin and plasminogen activator molecules. This inhibition prevents the conversion of plasminogen to plasmin, which is responsible for degrading fibrin clots [1]. As a result, TXA can effectively stabilize formed clots and impede the breakdown of fibrin [2].

TXA has been proven effective in reducing postoperative bleeding and transfusion rates in various procedures, such as cardiac surgery, liver transplantation, and cesarean section [3–5]. As for orthopedic surgery, administration of TXA has been widely utilized in total hip/knee arthroplasty and spinal procedures[6]. The use of TXA is progressively expanding to arthroscopic operations, including anterior cruciate ligament reconstruction (ACLR) procedures. Karaaslan et al. first demonstrated that intravenous TXA was associated with a decreased amount of postoperative hemarthrosis, decreased pain scores, and a reduced need for aspiration of the operated knee after ACLR [7]. The idea of intra-articular administration of TXA was first proposed by Chiang et al., who observed a significant reduction in postoperative drain volume and hemarthrosis grade compared with the placebo group [8]. Similar efficacies of both intravenous administration and intra-articular injection of TXA administration in reducing postoperative hemarthrosis, joint pain, and swelling following ACLR were demonstrated by Ma et al. [9].

While ACLR is generally regarded as a safe and minimally invasive procedure, there exist potential complications when intra-articular injection of TXA is administered. Several in vitro studies have reported that TXA may exhibit toxicity toward intra-articular cells, including chondrocytes, tenocytes, and osteoblast‐like cells [10]. Moreover, prior research has also suggested that the cytotoxicity effect of TXA is dose- and time-dependent [11]. Nevertheless, to date, the optimal regimen and protocol of TXA injection in ACLR remain elusive.

The study is companion research to a previous work [8]. We aimed to evaluate the effect of intra-articular injection of TXA and its acting time in patients undergoing arthroscopic ACLR. It is hypothesized that a longer acting time of the TXA injection might decrease postoperative drainage volume and pain scores.

## Patients and methods

In the current study, 150 patients receiving arthroscopic ACLR with autologous hamstring grafts between September 2023 and January 2024 were enrolled. Of these patients, 20 were excluded for the following reasons: previous knee procedures on the same side (*n* = 6), renal diseases (*n* = 3), coagulation disorders (*n* = 2), or refusal of participation in the study (*n* = 9). All the others provided signed informed consent for their participation. On the day of the operation, an independent investigator performed permuted block randomization using the blockrand package in R version 4.3.0 to divide the 130 patients into two groups: patients receiving intra-articular injection of TXA after ACLR with drainage clamped for 4 h (TXA 4 h group, *n* = 65) and 8 h (TXA 8 h group, *n* = 65), respectively. The study was approved by the Institutional Review Board of Taipei Veternas General Hospital (2023-09-017A).

### Surgical technique

All patients participating in this study underwent surgery performed by three fellowship-trained surgeons (HLM, ERC, and KHC), with spinal anesthesia administered under the same protocol. A tourniquet was routinely applied during the operation. A 3-cm long surgical wound was made on the medial aspect of the proximal tibia for access, and the semitendinosus and gracilis tendons were harvested. These tendons were then folded in a quadrupled fashion and secured using no. 2 and no. 5 Ethibond sutures (Ethicon, Somerville, NJ), as previously described [12]. Femoral sockets were created by drilling from the anatomical footprint on the lateral femoral condyle via the anteromedial portal. The tibial tunnel was prepared using an Acufex guide (Smith & Nephew, Andover, MA), ensuring the socket diameters met the size of the tendon graft. The grafts were then fixed with BioRCI-HA bioscrews (Smith & Nephew, Andover, MA) at both the femoral and tibial locations, with the knee flexed at 30 °. The sizes of the screw and tunnel were identical. The subsequent meniscus repair or meniscectomy was performed on the basis of the type and location of the meniscus tear, if necessary. Tears occurring in the red–red or red–white zones were treated with meniscus repair using the all-inside technique and a meniscus fixator (Zimmer, Warsaw, IN), whereas tears in the white zone or flap tears were managed with a meniscectomy. After the procedure, a Hemovac suction drain (Zimmer, Warsaw, IN) was introduced at the superior-lateral aspect of the joint. A total of 10 mL of TXA (100 mg/mL; Daiichi Sankyo, Tokyo, Japan) was then injected into the joint before the release of the tourniquet. The closed suction drains were clamped for either 4 h or 8 h, on the basis of the assigned group.

### Historical control

Two additional groups of patients were retrospectively retrieved from a previous study [8] for more comprehensive comparisons. These patients received either a placebo (placebo group) or a TXA injection after ACLR and had their drains clamped for 2 h (TXA 2 h group), serving as historical controls. Of note, the patients included in the current study were selected on the basis of the same criteria and followed identical surgical protocols, data collection methods, outcome assessments, and final analyses as those used in the previous study.

### Outcome assessment

Drain tubes were typically removed on postoperative day 2, 24 h after surgery, unless the drainage volume remained high. The total drainage volume at the 24 h mark was recorded as the primary outcome. Secondary outcomes included the visual analog scale (VAS), hemarthrosis grade (Coupens and Yates), and the International Knee Documentation Committee (IKDC) functional score. VAS scores from 0 (no pain) to 10 (worst pain possible) were recorded using a traditional paper-based item for each patient immediately after being transferred from the postanesthesia recovery room to the ward on day 3 and at week 4 postsurgery [13]. The highest VAS score recorded during the postoperative admission was also noted. Hemarthrosis was graded from 0 (no detectable fluid) to 4 (tense hemarthrosis) on day 3 and at week 4 [14]. The IKDC functional score was assessed through a subjective questionnaire evaluating symptoms, athletic activity, and knee function, with scores ranging from 0 (worst) to 10 (best) [15]. All objective measurements were reviewed by an independent, blinded observer (TJH) at the outpatient clinic.

### Statistical analysis

Power analysis was performed using G*Power software (version 3.1; Heinrich-Heine-Universität Düsseldorf, Düsseldorf, Germany). On the basis of the volume of drainage observed in a previous study, a sample size of 49 patients per group was calculated, assuming a power of 0.8 and a significance level of 0.05. To mitigate the impact of potential withdrawals and loss to follow-up, oversampling was implemented. Data are presented as means with 95% confidence intervals or standard deviations. Independent *t*-tests were used to compare drainage volumes, VAS scores, and IKDC scores between groups. Hemarthrosis grades were evaluated with the chi-squared test. For comparisons among the four groups regarding drainage amounts, VAS scores, and IKDC scores, analysis of variance (ANOVA) with post hoc Tukey Honestly Significant Difference tests were employed. For the univariable and multivariable analyses, a linear regression model was employed to evaluate the effects of TXA use, drainage clamping duration, and other factors that might influence postoperative drainage volume. Variables found to be statistically significant in the univariable analysis were subsequently included in the multivariable analysis. The results are presented as odds ratios with corresponding 95% confidence intervals. Statistical analyses were conducted using SPSS software (version 22.0; IBM, Armonk, NY), with significance set at *P* < 0.05.

## Results

**Fig. 1:**
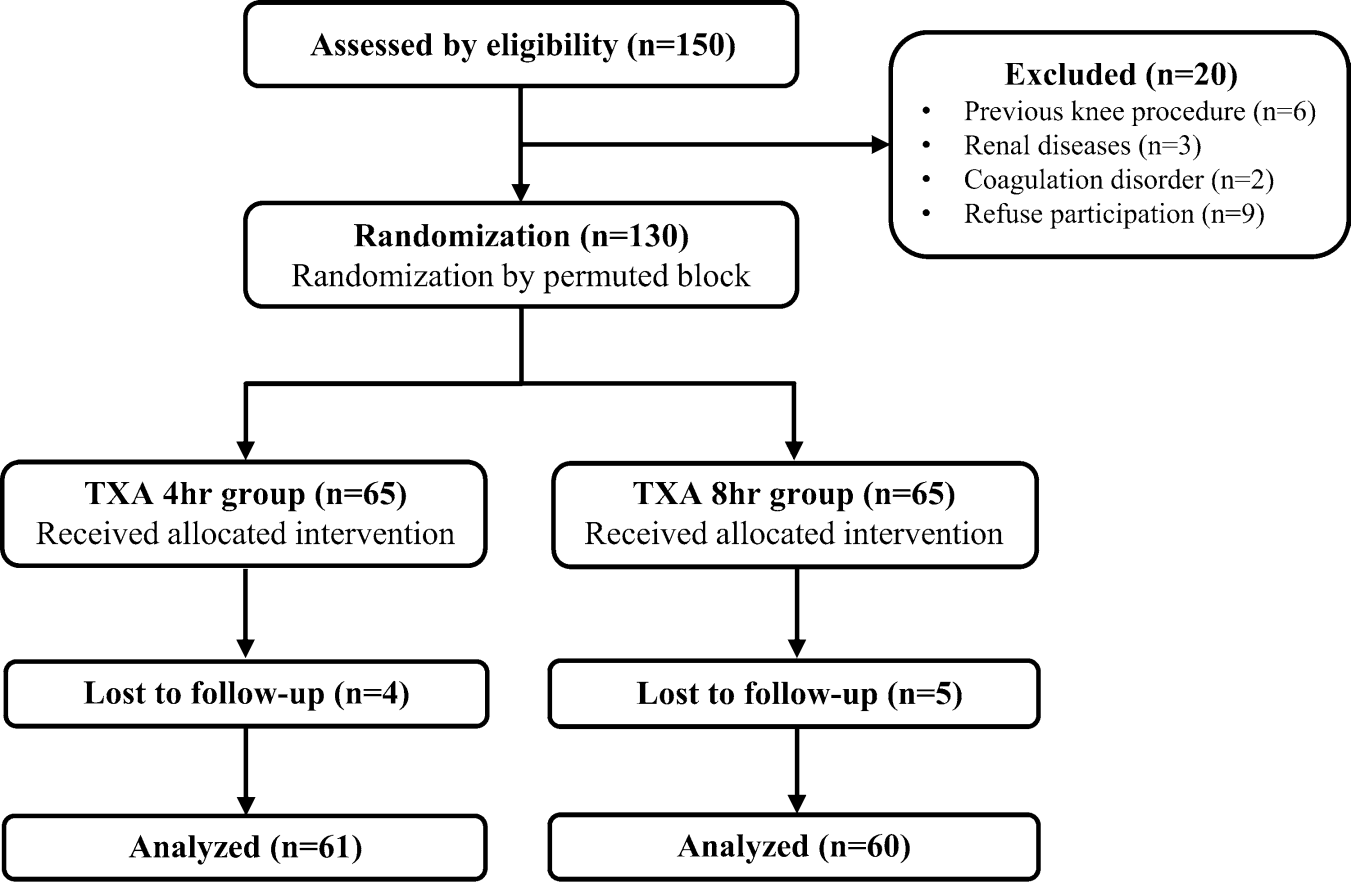
Flowchart of enrolled patients; TXA, tranexamic acid

Of the 130 patients included, 121 completed the follow-up examination and were categorized into the TXA 4 h group (*n* = 61) and the TXA 8 h group (*n* = 60) (Fig. 1). Detailed demographic characteristics of the included patients are listed in Table 1.

**Table 1:** Patient demographic characteristics

Variable	TXA 4 h	TXA 8 h
No. of patients	61	60
Age [years]	31.1 ± 8.9	32.1 ± 8.6
Sex, *n* (%)		
Female	21 (34.4%)	18 (30.0%)
Male	40 (65.6%)	42 (70.0%)
Pre-operative IKDC function score	4.5 ± 2.1	4.4 ± 2.4
Procedure type		
ACLR, *n* (%)	19 (31.1%)	21 (35.0%)
ACLR + PM, *n* (%)	19 (31.1%)	5 (8.3%)
ACLR + meniscus repair, *n* (%)	23 (37.7%)	34 (56.7%)
Average operation time, min	53.8 ± 13.5	57.1 ± 12.2

Data are presented as means ± standard deviations, unless otherwise indicated

TXA, tranexamic acid; IKDC, International Knee Documentation Committee; ACLR, anterior cruciate ligament reconstruction; PM, partial meniscectomy

No significant change in drainage was observed among patients who received intra-articular TXA injections with the suction drain clamped for 4 h or 8 h **(**Table 2: TXA 4 h group: 39.9 ± 10.4 mL; TXA 8 h group: 34.9 ± 7.0 mL; *P* = 0.8989). In the subgroup analysis, the postoperative drainage of patients undergoing either isolated ACLR or the additional procedures including meniscal repair or meniscectomy did not show significant differences between the two groups either.

**Table 2:** Postoperative drainage output in different meniscal procedures

	TXA 4 h, mL	TXA 8 h, mL	*P*-value
Total patients	39.9 (29.5–50.3)	34.9 (27.8–41.9)	0.8989
ACLR only	41.1 (25.3–56.8)	31.9 (23.5–40.3)	0.4056
ACLR + PM	46.1 (16.9–75.4)	60.0 (17.9–102.1)	0.4162
ACLR + meniscus repair	34.1 (22.5–45.8)	32.7 (22.1–43.2)	0.9971

Data are presented as means (95% confidence interval)

ACLR, anterior cruciate ligament reconstruction; mL, milliliter; PM, partial meniscectomy; TXA, tranexamic acid

On postoperative day 3, significantly decreased grades of hemarthrosis were noted in the TXA 8 h group (*P* = 0.0300); however, no differences were observed at the week 4 follow-up (Table 3). There were no significant differences in VAS scores at different postoperative time points or in IKDC scores at 4 weeks postoperative between the TXA 4 h and the TXA 8 h groups. No complications, such as deep vein thrombosis, knee infection, or arthrofibrosis, were reported in both groups.

**Table 3:** Clinical results

	TXA 4 h (*n* = 61)	TXA 8 h (*n* = 60)	*P*-value
Hemarthrosis, grade 0/1/2/3/4, *n*			
Day 3	12/29/13/5/2	25/27/5/2/1	0.0300
Week 4	39/13/5/3/0	44/13/3/0/0	0.6380
VAS score			
Initial after operation	2.5 ± 0.8	2.7 ± 1.8	0.8607
Max during admission	5.5 ± 1.5	5.3 ± 1.8	0.9457
Day 3	3.0 ± 0.5	2.7 ± 0.6	0.7432
Week 4	1.0 ± 1.3	1.1 ± 1.3	0.9494
IKDC function score (0–10) (week 4)	7.4 ± 2.0	6.9 ± 1.6	0.2310

Data are presented as means ± standard deviations, unless otherwise indicated

TXA, tranexamic acid; VAS, visual analog scale; IKDC, International Knee Documentation Committee

**Fig. 2:**
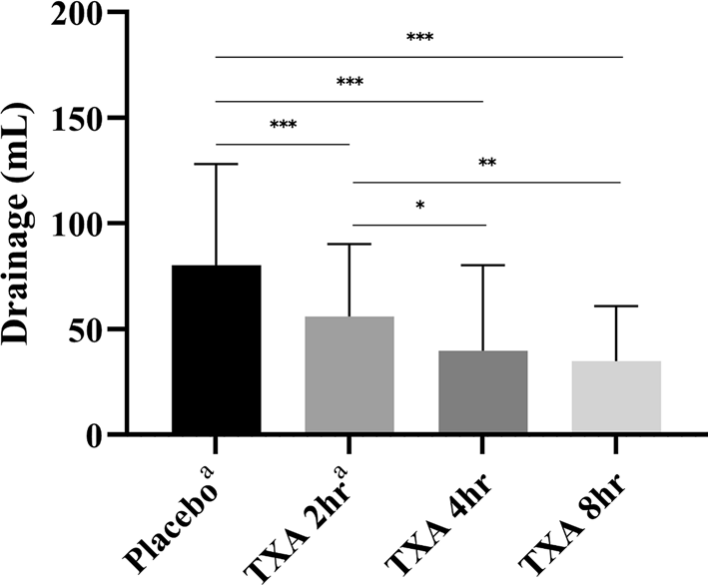
Volume of drainage output 24 h postoperatively; *P < 0.05; **P < 0.01; ***P < 0.001; ^a^data from the placebo group and TXA 2 h group were retrieved from Chiang et al. [8] mL, milliliter; TXA, tranexamic acid

Two groups of patients from a previous study, the TXA 2 h group (*n* = 151) and the placebo group (*n* = 149), were included as historical controls for further comparisons of postoperative drainage amounts, VAS scores, and IKDC scores. Significant reductions in drainage were observed among patients who received postoperative intra-articular TXA injections with the suction drain clamped for either 4 h or 8 h compared with the placebo and TXA 2 h groups (*P* < 0.05 between groups). (Fig. 2). In addition, multivariate analysis revealed that the use of TXA and longer clamping durations were independently associated with reduced drainage volumes, as indicated by the linear regression model (Table 4).

**Table 4:** Univariate and multivariate analysis of factors affecting drainage volume post-ACLR

Variable	Univariate, OR (95% CI)	Multivariate, OR (95% CI)
Use of TXA (reference: placebo)	* −3.622 (−2.791, −4.454)*	* −3.323 (−2.480, −4.166)*
Drainage clamping time (reference: 2 h)	* −2.905 (−1.936, −3.874)*	* −1.053 (−0.311, −1.795)*
Age	−0.135 (0.245, −0.516)	
Sex (reference: female)	*1.295 (2.032, 0.559)*	0.342 (1.385, −0.701)
Pre-operative IKDC function score	−0.954 (2.223, −4.131)	
Procedure type (reference: ACLR alone)	3.292 (7.874, −1.291)	
Operating time	0.101 (0.379, −0.177)	

Data are presented as means ± standard deviations, unless otherwise indicated

ACLR, anterior cruciate ligament reconstruction; CI, confidence interval; IKDC, International Knee Documentation Committee; OR, odds ratio; TXA, tranexamic acid

**Fig. 3:**
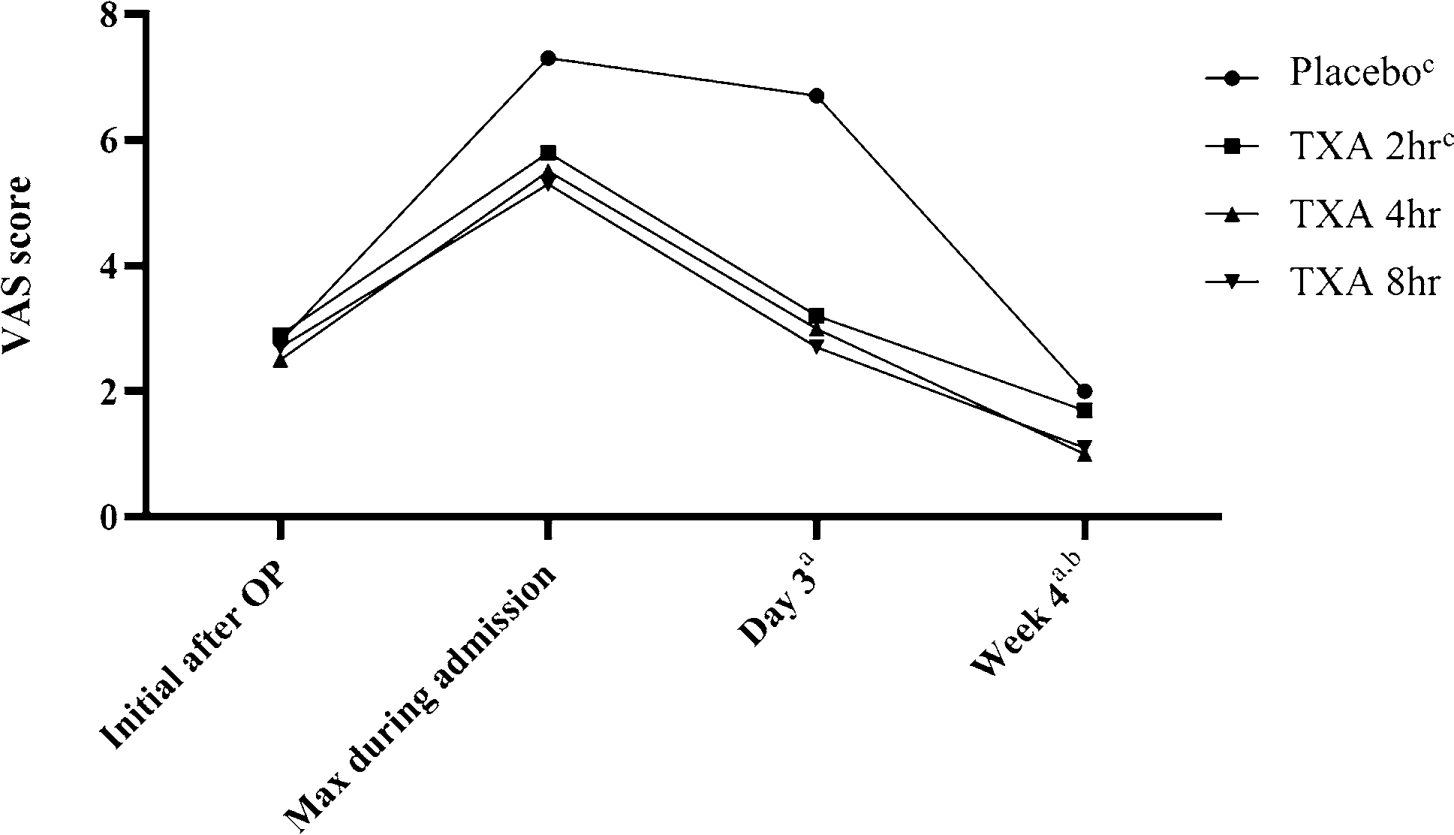
Mean of VAS score at different postoperative periods; ^a^*P* < 0.05 between groups of placebo and TXA 2 h, placebo and TXA 4 h, and placebo and TXA 8 h; ^b^*P* < 0.05 between groups of TXA 2 h and TXA 4 h, TXA 2 h and TXA 8 h; ^c^data from the placebo group and the TXA 2 h group were retrieved from Chiang et al. [8] OP, operation; max, maximum; TXA, tranexamic acid; VAS, visual analogue scale

**Fig. 4:**
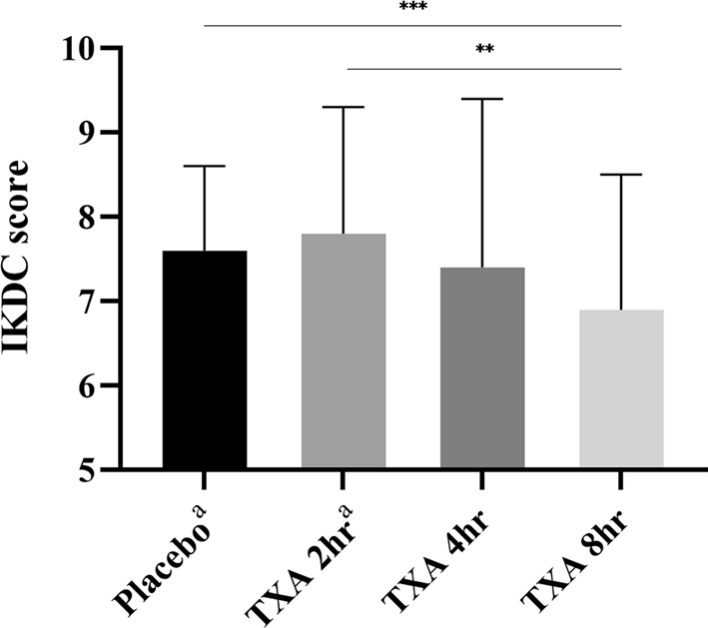
IKDC score measured at week 4 postoperatively; **P < 0.01; ***P < 0.001; ^a^data from the placebo group and the TXA 2 h group were retrieved from Chiang et al. [8] IKDC, International Knee Documentation Committee; TXA, tranexamic acid

The results of the VAS scores on postoperative day 3, at week 4, and the maximum postoperative VAS score during admission demonstrated a significant reduction in the TXA 2 h, TXA 4 h, and TXA 8 h groups when compared with the placebo group (Fig. 3). However, only the VAS scores at 4 weeks postoperative showed a significant decrease in the TXA 4 h and TXA 8 h groups in comparison with the TXA 2 h group (*P* < 0.001). Of note, the IKDC scores were significantly worse in patients with TXA clamping for 8 h compared with those with clamping for only 2 h (*P* < 0.001) and the placebo group (*P* = 0.009) (Fig. 4).

## Discussion

To the best of our knowledge, this is the first study discussing the effects of different acting times of intra-articular TXA injection on postoperative drainage volume, hemarthrosis, pain scores, and functional outcomes. The key findings of this study are the following: (1) intra-articular injection of TXA, with drainage clamping for either 4 h or 8 h, led to a similar decrease in postoperative drainage, VAS score, and IKDC score; (2) intra-articular TXA injection, with drainage clamping for 8 h, resulted in a significant reduction in hemarthrosis on postoperative day 3 compared with those with clamping for 4 h; (3) although drainage clamping for either 4 h or 8 h resulted in similar IKDC scores in our study, prolonged drainage clamping for 8 h following intra-articular TXA injection was associated with a significant worsening of IKDC scores at week 4 compared with the placebo group and the TXA 2 h group, according to data from previous studies.

The overall complication rate for various arthroscopic knee procedures was documented to be as high as 1.68% [16–19]. Hemarthrosis, constituting the predominant factor in 60.1% of all complications, might pose a significant risk for postoperative pain, swelling, and decreased range of motion (ROM) during the early postoperative period. The use of conservative prophylactic measures, such as hemovac drains, was proven to decrease postoperative hemarthrosis in ACLR [14]. Despite these preventive measures, Andrés-Cano et al. still reported that 13.2% of patients post-ACLR sought emergency department care postdischarge, with pain (6.7%) and tension hemarthrosis necessitating arthrocentesis (4.4%) emerging as the most prevalent complications [20]. Recently, TXA has been widely utilized to decrease perioperative bleeding and postoperative hemorrhage in multiple interventions, including orthopedic and spinal surgeries [21, 22]. As for arthroscopic procedures, Li et al. conducted a study on knee arthroscopic arthrolysis and found that patients who were administered topical TXA had significantly reduced drainage volume compared with those who did not receive TXA injection [23]. Moreover, lower VAS pain scores and better Lysholm knee scores were also observed at week 1 and week 2 postoperatively in patients receiving topical TXA injection. Another systematic review and meta-analysis, which included seven RCTs, demonstrated that TXA use significantly improves VAS scores up to 6 weeks postoperatively, reduces drainage output, and decreases incidences of joint aspiration and hemarthrosis grades (Coupens and Yates) in various arthroscopic procedures such as ACLR, meniscectomy, femoroacetabular impingement incision, and rotator cuff repair [24].

There is a growing body of evidence supporting the use of TXA in ACLR. Alkhatib et al. pooled 807 patients undergoing ACLR from seven RCTs [25]. The results of the meta-analysis demonstrated that the VAS score of patients receiving ACLR with TXA injection was significantly decreased at 2 weeks and 3–6 weeks postoperatively. Hemarthrosis grades (Coupens and Yates), drain output, and knee swelling in the initial postoperative period were also reduced in those treated with TXA. In the subgroup analysis, the phenomenon of decreased drainage volume, VAS score at 3–6 weeks postoperatively, and hemarthrosis grades (Coupens and Yates) was also observed in patients with intra-articular TXA administration compared with the placebo group. These findings are consistent with previous results, indicating correlation of TXA injection with reduced drainage volume and lower maximum VAS scores on postoperative day 3 and at 4 weeks. In the current study, with prolonged acting time to enhance the efficacy of TXA in the joint space, we also observed a significant reduction in drainage output and VAS scores at postoperative week 4 between the TXA 2 h and TXA 4 h/8 h groups. These results indicate a prominent analgesic effect of TXA in the early stages of recovery, which might be beneficial to postoperative mobilization and rehabilitation.

The routine use of a closed suction drain system in ACLR has been a topic of debate for a long time [26]. Clifton et al. first argued that closed suction surgical wound drainage was unnecessary for ACLR procedures since no significant differences in postoperative range of motion or functional scores were observed in a systematic review [27]. However, this study did not account for scenarios involving the concurrent use of TXA. Nevertheless, despite merits of topical TXA injection, including reduced postoperative drainage volume, lower hemarthrosis levels, and alleviated VAS scores, one of the considerations is the potential cytotoxicity of TXA toward intra-articular cells. Research indicates that these cytotoxic effects are largely dose-dependent [28–30]. A recent in vivo study using a rat model demonstrated that intra-articular injection of TXA at concentrations of 100 mg/mL or higher may reduce cell viability in both cartilage and the meniscus, leading to significant cartilage degeneration in rats following anterior cruciate ligament (ACL) transection procedures [31]. Notably, there are currently no studies investigating the harmful effects of TXA on human chondrocytes in vivo. In addition, these effects, in relation to topical TXA, have not been reported in arthroplasty or spinal surgeries. Moreover, a recent histological study found that intra-articular administration of TXA was more protective of articular cartilage and ACL integrity than intravenous injection in an experimental rat model [32]. In our study, we observed a significant decrease in IKDC scores at 4 weeks postoperatively in the TXA 8 h group compared with the TXA 2 h group, but no obvious benefits regarding postoperative drainage and pain level between groups clamping for 4 h and 8 h. These findings suggest that a 4 h acting duration, followed by intra-articular injection of 10 mL of TXA (100 mg/mL), may be an efficient and safe approach in routine ACLR procedures. However, the detailed mechanisms by which TXA affects cartilage and tendon in human bodies, as well as its potential impact on long-term functional outcomes, still warrant further investigation.

Several limitations of this study should be acknowledged. First, the relatively short follow-up period of 4 weeks postoperatively may not capture long-term outcomes and complications that could be associated with TXA administration. Second, parts of the analysis involved patient data collected from a previous randomized study. This might have introduced potential confounding factors despite efforts to minimize bias and ensure randomization in the current study. Lastly, owing to the inability to blind patients to the clamping time, there is a possibility of bias influencing the results. Furthermore, prolonged closure of the drainage system could lead to increased intra-articular pressure, potentially confounding the true efficacy of TXA in reducing drainage volume.

## Conclusions

This study provides further evidence supporting the efficacy of intra-articular TXA injection in patients undergoing arthroscopic ACLR. A 4 h clamping time for TXA administration after ACLR may be considered in current practice, as it effectively reduces drainage and pain without negatively impacting functional outcomes.

## Data Availability

The datasets generated and/or analyzed during this study are available from the corresponding author on reasonable request.

## Abbreviations

ACLR: Anterior cruciate ligament reconstruction
IKDC: International Knee Documentation Committee
TXA: Tranexamic acid
VAS: Visual analog scale
